# The Periventricular Nucleus as a Brain Center Containing Dopaminergic Neurons and Neurons Expressing Individual Enzymes of Dopamine Synthesis

**DOI:** 10.3390/ijms23126739

**Published:** 2022-06-16

**Authors:** Michael V. Ugrumov, Ekaterina N. Pavlova, Anna A. Kolacheva, Liliya K. Dil’mukhametova, Vsevolod V. Bogdanov, Victor Blokhin, Tatiana S. Pronina

**Affiliations:** Laboratory of Neural and Neuroendocrine Regulations, Koltzov Institute of Developmental Biology of the Russian Academy of Sciences, Moscow 119334, Russia; guchia@gmail.com (E.N.P.); annakolacheva@gmail.com (A.A.K.); lili.dilm@gmail.com (L.K.D.); vse-bogd@yandex.ru (V.V.B.); victor.blokhin@hotmail.com (V.B.); tatiana.pronina@mail.ru (T.S.P.)

**Keywords:** hypothalamus, periventricular nucleus, dopaminergic neuron, dopamine, L-DOPA, tyrosine hydroxylase, aromatic L-amino acid decarboxylase, third cerebral ventricle, cerebrospinal fluid, rat

## Abstract

Since the 1980s, the concept of dopamine-rich brain centers as clusters of only dopaminergic neurons has been fundamentally revised. It has been shown that, in addition to dopaminergic neurons, most of these centers contain neurons expressing one of the enzymes of dopamine synthesis: tyrosine hydroxylase (TH) or aromatic L-amino acid decarboxylase (AADC). We have obtained convincing evidence that in rats, the hypothalamic periventricular nucleus (PeVN) is one of the largest dopamine-rich centers, containing dopaminergic and monoenzymatic neurons. Indeed, using double immunostaining for TH and AADC, the PeVN was shown to contain almost three thousand dopaminergic and monoenzymatic neurons. According to high-performance liquid chromatography, PeVN contains L-DOPA and dopamine, which, apparently, are synthesized in monoenzymatic TH neurons and bienzymatic neurons, respectively. According to confocal microscopy, neurons (cell bodies, fibers), which were immunopositive only to TH, only to AADC, or both, are in close topographic relationships with each other and with the 3rd ventricle. These data suggest the mutual regulation of the neurons, as well as the delivery of dopamine and L-DOPA to the third ventricle, which is confirmed by their detection in the cerebrospinal fluid. Thus, evidence has been obtained that PeVN is one of the largest dopamine-rich centers of the brain, containing dopaminergic and monoenzymatic neurons.

## 1. Introduction

The development of the histofluorescence method for the detection of catecholamines in combination with spectroscopy in the 1960s and later that of the immunohistochemical method for the detection of tyrosine hydroxylase (TH), the first rate-limiting enzyme for the synthesis of catecholamines, made it possible to identify the brain centers that produce catecholamines and to map their location [1,2,3]. It has been shown that the most numerous brain centers of this kind contain dopamine and TH-expressing neurons [1,2,3,4]. Proceeding from these data, it has long been believed that TH-immunopositive neurons detected by monoimmunolabeling are dopaminergic neurons synthesizing dopamine from L-tyrosine, a precursor amino acid.

The concept of dopaminergic centers of the brain has been significantly revised after the development of an immunohistochemical method for detecting aromatic L-amino acid decarboxylase (AADC), the second enzyme of dopamine synthesis, as well as for the double immunolabeling of both enzymes. This has made it possible to show that a number of such brain centers, for example, the arcuate nucleus of the hypothalamus, in addition to dopaminergic neurons expressing both enzymes of dopamine synthesis, contain so-called monoenzymatic neurons expressing only TH or only AADC [5,6,7,8,9]. Moreover, convincing evidence has been obtained that the final synthetic product in monoenzymatic TH neurons is L-DOPA, and in monoenzymatic AADC neurons it is dopamine [5,10,11].

Surprisingly, despite the obtained evidence that TH is expressed not only in bienzymatic (dopaminergic) neurons, but also in monoenzymatic (non-dopaminergic) neurons, in a number of studies, neurons with only TH detected with monoimmunolabeling are still considered to be dopaminergic [12,13,14].

Over the past two decades, many studies have focused on the functioning of monoenzymatic neurons, mainly in the arcuate nucleus (AN) of the hypothalamus. In these studies, some fundamentally important data were obtained. First, it was shown that the number of monoenzymatic neurons in the AN in adult animals significantly exceeds that of bienzymatic neurons, which indirectly indicates their important functional significance [15]. Second, evidence was obtained that monoenzymatic neurons expressing complementary enzymes for dopamine synthesis synthesize dopamine in cooperation. This means that L-DOPA synthesized from L-tyrosine in monoenzymatic TH neurons is released into the intercellular space and captured by nearby monoenzymatic neurons expressing AADC, where dopamine is synthesized [16,17]. It is assumed that L-DOPA, in addition to participating in the cooperative synthesis of dopamine, plays the role of a neurotransmitter [18,19,20,21]. Thirdly, it was shown that monoenzymatic neurons appear earlier in ontogenesis than bienzymatic neurons, which indicates their special role in the critical period of morphogenesis [15]. It is assumed that during this period of development, L-DOPA that is secreted by monoenzymatic TH neurons plays the role of a morphogenetic or transcription factor [9]. Fourth, the number of monoenzymatic neurons and the amount of dopamine synthesized by these neurons increase with the degeneration of dopaminergic neurons. This is considered to be an important mechanism of brain neuroplasticity in neurodegenerative diseases, mainly Parkinson’s disease (PD) and hyperprolactinemia [8,9,22].

Over the last 20 years, monoenzymatic neurons have been found throughout the brain [9,23]. In addition to the hypothalamus, monoenzymatic neurons have been found in the substantia nigra, the main dopaminergic center of the brain, in various mammals, including primates (humans) [7,24,25,26]. Most studies emphasize that the number of monoenzymatic neurons is extremely small when compared to that of bienzymatic dopaminergic neurons. In addition to the brain, monoenzymatic neurons are also widespread in the spinal cord [27,28].

Given the fact that occasional neurons containing the enzymes of dopamine synthesis were described in the PeVN, as in the AN, and both nuclei are located in close vicinity and play a key role in the neuroendocrine regulation of reproduction [12,29,30,31,32,33], we hypothesized that the PeVN, similar to the AN, is a dopaminergic center containing numerous monoenzymatic and bienzymatic neurons. Therefore, the aim of the present study was to test this hypothesis by accomplishing the following objectives: (1) to obtain evidence that the PeVN contains a significant number of bienzymatic (dopaminergic) neurons and monoenzymatic neurons expressing only TH or only AADC, and to quantify each of the populations; (2) to determine if the PeVN contains L-DOPA as the final synthetic product in monoenzymatic TH neurons, and dopamine as the final product in bienzymatic and monoenzymatic AADC neurons; (3) if monoenzymatic TH and AADC neurons are found in the PeVN, to test whether they synthesize dopamine in co-operation; (4) to study the topographic relations of monoenzymatic and bienzymatic neurons in the PeVN and within the third cerebral ventricle; (5) to determine whether the cerebrospinal fluid contains L-DOPA and dopamine as an indicator of the secretion of L-DOPA by monoenzymatic TH neurons and dopamine by monoenzymatic AADC neurons and bienzymatic (dopaminergic) neurons; (6) to carry out a comparative analysis of the PeVN and AN as dopaminergic centers of the brain containing dopaminergic (bienzymatic) and monoenzymatic TH and AADC neurons, using the data obtained in this and previous studies.

## 2. Results

### 2.1. Dopamine and L-DOPA Levels in the Cerebrospinal Fluid and in the Periventricular and Arcuate Nuclei

The dopamine concentration in the cerebrospinal fluid (CSF) was 0.59 ± 0.05 pmol/mL, while the L-DOPA concentration was 1.3 ± 0.07 pmol/mL.

**Figure 1:**
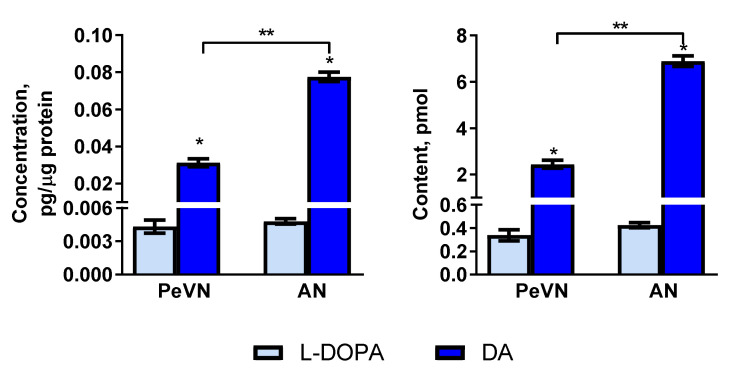
Content and concentration of L-DOPA and dopamine (DA) in the periventricular nucleus (PeVN) and arcuate nucleus (AN) of rats. * *p* < 0.05, significant differences between L-DOPA and dopamine levels; ** *p* < 0.05, significant differences between selected parameters.

The dopamine content in the PeVN was 2.44 ± 0.17 pmol, while the dopamine concentration in the same location was 0.031 ± 0.002 pmol/μg protein. The content and concentration of dopamine in the AN were higher than in the PeVN by 2.8 and 2.3 times, respectively (Figure 1). In contrast to the dopamine content, the content and concentration of L-DOPA in the PeVN (0.34 ± 0.05 pmol and 0.0043 ± 0.0006 pmol/μg protein, respectively) did not differ from the content and concentration of L-DOPA in the AN (0.43 ± 0.02 pmol and 0.0048 ± 0.0002 pmol/μg protein, respectively) (Figure 1).

### 2.2. Dopamine Content in Vibratome Sections of the Periventricular Nucleus, Arcuate Nucleus, and Substantia Nigra and in the Incubation Medium after Incubating the Sections with 2-Amino-2-Norbornanecarboxylic Acid, as Well as in the Control

**Figure 2:**
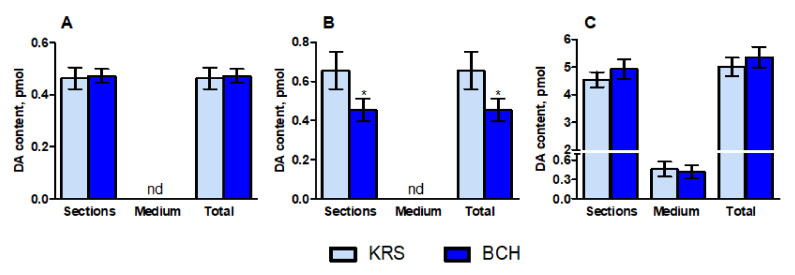
Dopamine content in vibratome sections of the periventricular nucleus (**A**), arcuate nucleus (**B**), and substantia nigra (**C**) after their incubation in Krebs–Ringer solution (KRS) in the presence or absence of 0.5 mM 2-amino-2-norbornanecarboxylic acid (BCH), and in the incubation medium, as well as the total dopamine content in the incubation medium and vibratome sections. * *p* < 0.05, significant differences in dopamine content after the incubation of vibratome sections with or without BCH; nd, not detectable.

Periventricular nucleus. The dopamine content in PeVN sections after incubation in Krebs–Ringer solution (KRS) was 0.46 ± 0.04 pmol. When PeVN sections were incubated with 0.5 mM 2-amino-2-norbornanecarboxylic acid (BCH), the content of dopamine did not change. No dopamine was detected in the incubation medium (Figure 2).

Arcuate nucleus. The dopamine content in AN sections after incubation in KRS was 0.66 ± 0.1 pmol. When AN sections were incubated with 0.5 mM BCH, the content of dopamine was reduced by 1.5 times. No dopamine was detected in the incubation medium (Figure 2).

Substantia nigra. The dopamine content in SN sections after incubation in KRS was 4.54 ± 0.27 pmol. When SN sections were incubated with 0.5 mM BCH, the content of dopamine did not change. In the incubation medium, the dopamine content was 0.46 ± 0.12 pmol, and did not change (Figure 2) with the addition of BCH.

### 2.3. Neurons That Are Immunopositive for Tyrosine Hydroxylase, Aromatic L-Amino Acid Decarboxylase, and Both Enzymes in the Periventricular Nucleus

Given that out of all the monoaminergic neurons, the hypothalamus and, in particular, the PeVN contain only dopaminergic neurons, it was sufficient to use double immunolabeling for TH and AADC to identify the cell bodies of neurons expressing the enzymes of dopamine synthesis. However, to assess the topographic relationships between neuron cell bodies and nerve fibers containing dopamine-synthesizing enzymes, it was necessary to use an antibody cocktail which would allow us to exclude other monoaminergic fibers from consideration, also containing TH (noradrenergic fibers) and AADC (serotonergic fibers).

**Figure 3:**
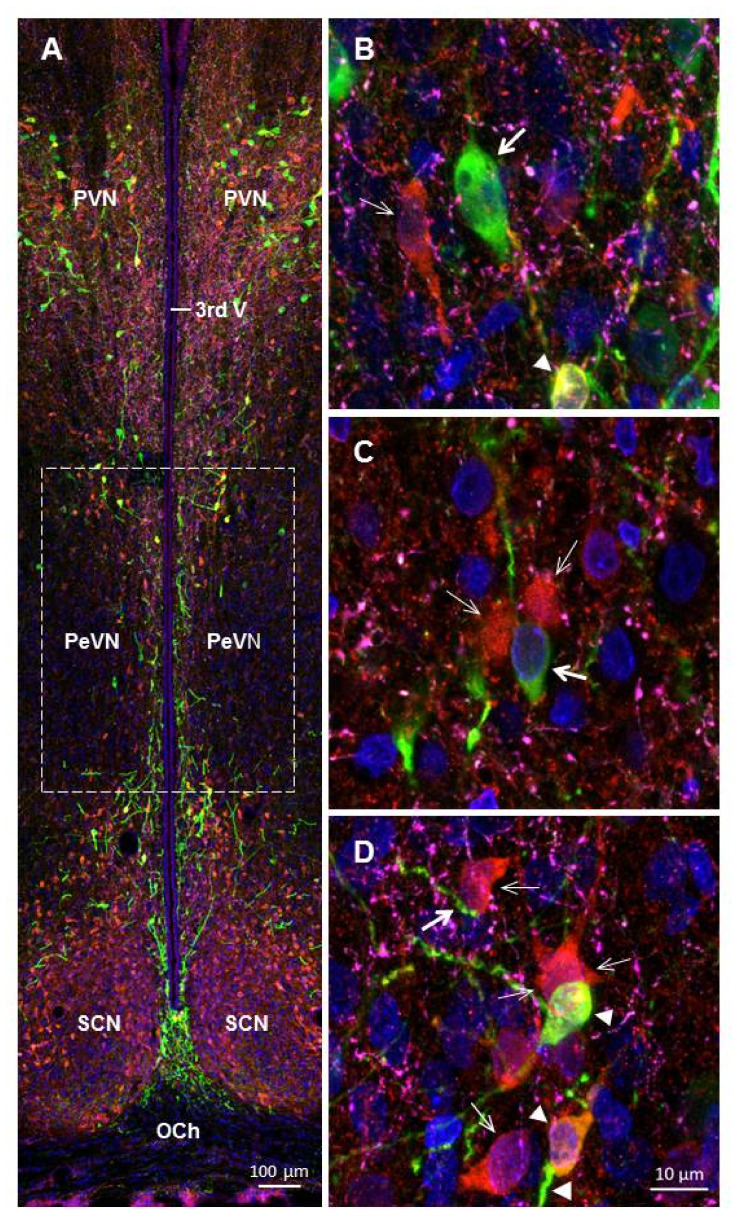
Confocal microscopy of the periventricular nucleus (PeVN) of the rat on frontal sections of the brain (**A**–**D**). (**A**) general view of the PeVN (dotted frame); (**B**,**C**) neurons, immunopositive for tyrosine hydroxylase (TH), but immunonegative for aromatic L-amino acid decarboxylase (AADC) (green, thick arrow); (**B**–**D**) neurons immunopositive for AADC, but immunonegative for TH (red, thin arrow); (**B**,**D**) neurons, immunopositive for TH and AADC (yellow, arrowhead). Scale: A 100 µm, B-D 10 µm. OCh, optical chiasma; PVN, paraventricular nucleus; SCN, suprachiasmatic nucleus; 3rd V, third ventricle.

The following types of neurons were found at all levels of the PeVN in the rostro-caudal direction: (i) TH-immunopositive (TH+), but AADC-immunonegative (AADC−); (ii) AADC-immunopositive (AADC+) and TH-immunonegative (TH−); (iii) TH-immunopositive and AADC-immunopositive (TH+/AADC+) (Figure 3). Most of the neurons were oval bipolar in shape. Multipolar neurons, mostly pyramid-like neurons with three processes, were much less common. All the neurons were located in topographic proximity to one another. Thus, contacts were found between the cell bodies of: AADC+/TH− neurons (Figure 3B), AADC−/TH+ neurons, and AADC+//TH− neurons (Figure 3C), as well as between the bodies of AADC+/TH− neurons and AADC+/TH+ neurons (Figure 3D).

**Figure 4:**
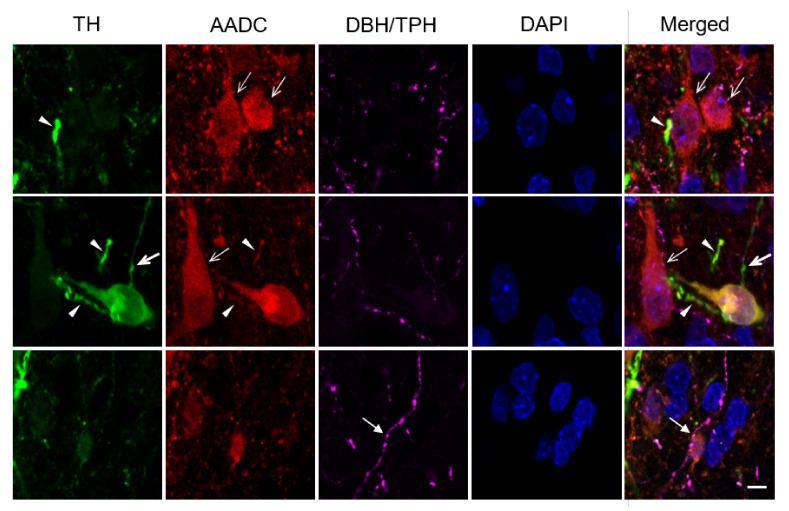
Observations in a confocal microscope of contacts of nerve fibers immunopositive only for tyrosine hydroxylase (TH+, green, thick arrow), only for aromatic L-amino acid decarboxylase (AADC+, red, thin arrow), for both enzymes (TH+/AADC+, yellow, arrowhead), as well as nerve fibers immunopositive for tryptophan hydroxylase (TPH) and/or dopamine-β-hydroxylase (DBH) (purple, closed arrow) with cell bodies and proximal processes of neurons TH+/AADC− (green), AADC+/TH− (red), and TH+/AADC− (yellow)) in the PeVN in rats. Scale: 5 µm; ×63.

The contacts between neuronal cell bodies and neuron proximal processes, on the one hand, and nerve fibers with varicose, probably synaptic terminals, on the other hand are of particular interest (Figure 4). Indeed, we have found all possible combinations of such contacts, in terms of immunopositivity for TH and AADC of the neuronal structures involved in their formation. It should be noted that not only axons immunopositive for dopamine-synthesizing enzymes, but also axons that are immunopositive for serotonin and / or DβH take part in such contacts (Figure 4).

**Figure 5:**
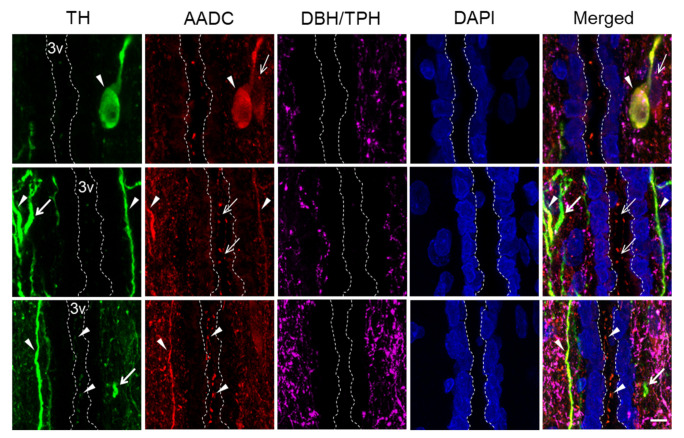
Observations in a confocal microscope of the topographic relations of neurons (cell bodies) and fibers, immunopositive only for tyrosine hydroxylase (TH+, green, thick arrow), only for aromatic amino acid decarboxylase (DAA+, red, thin arrow), for both enzymes (TH+/DAA+, yellow, arrow head), as well as nerve fibers immunopositive for tryptophan hydroxylase (TPH) and/or dopamine-β-hydroxylase (DBH) (purple, closed arrow) within the 3rd ventricle. All types of neurons and fibers are located in the subependymal zone, whereas some fibers, immunopositive for both enzymes or only for AADC, are seen between the ependymal cells and in the 3rd ventricle. Scale: 5 µm; ×63. 3v, third ventricle; dotted line, apical surface of the ependymal lining facing the cerebrospinal fluid.

Of no less interest are our data on the close topographic relationships of nerve fibers with the third ventricle (Figure 5). Thus, AADC+/TH− fibers were found in the lumen of the ventricle, between the ependymal cells and in the subependymal zone (Figure 5). In much smaller numbers and only in the subependymal zone, TH+/AADC− and TH+/AADC+ nerve fibers, as well as nerve fibers immunopositive for serotonin and/or DβH, were found (Figure 5).

**Figure 6:**
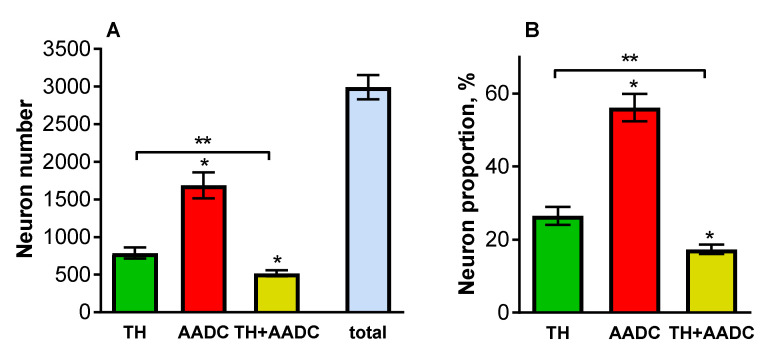
Quantification of neurons in the periventricular nucleus (PeVN), immunopositive for tyrosine hydroxylase (TH) only, aromatic L-amino acid decarboxylase (AADC) only, and both dopamine-synthesizing enzymes—TH and AADC. (**A**). The number of neurons in each of the populations of immunopositive neurons and the total number of all immunopositive neurons; (**B**). The proportion of each of the populations of immunopositive neurons in relation to the total number of immunopositive neurons, taken as 100%. * *p* < 0.05, significant differences between the previous and subsequent neuron populations; ** *p* < 0.05, significant differences between the selected neuron populations.

Quantification of the neurons containing dopamine-synthesizing enzymes showed that the PeVN contains 2993 ± 162 TH+/AADC−, TH−/AADC+, and TH+/AADC+ neurons. Among them, AADC+/TH− neurons were the most numerous (1688 ± 172) (Figure 6A). There were significantly fewer TH+/AADC− neurons (788 ± 73). Finally, the smallest population was represented by TH+/AADC+ neurons (517 ± 41) (Figure 6A). If we take the total number of neurons that were immunopositive for dopamine synthesis enzymes as being 100%, then the populations of TH+/AADC−, TH−/AADC+, and TH+/AADC+ neurons would amount to 26.48, 56.16, and 17.36%, respectively (Figure 6B).

**Figure 7:**
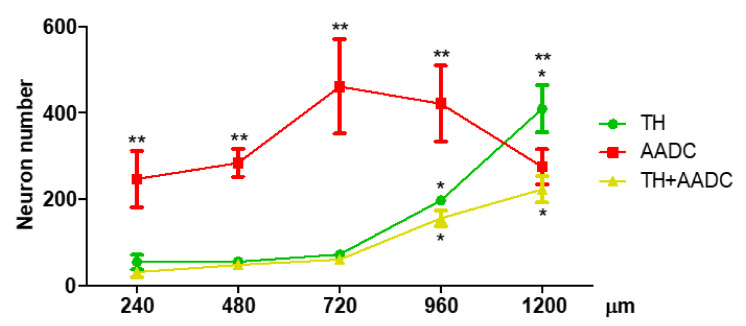
Number of neurons that were immunopositive for tyrosine hydroxylase (TH) only, aromatic L-amino acid decarboxylase (AADC) only, and both dopamine-synthesizing enzymes—TH and AADC, at various frontal levels of PeVN in the rostro-caudal direction. * *p* < 0.05, significant differences in the number of immunopositive neurons of each population at the adjacent frontal levels of the PeVN in the rostro-caudal direction; ** *p* < 0.05, significant differences between the number of immunopositive neurons of different populations at the same frontal level of PeVN in the rostro-caudal direction.

All the populations of neurons—TH+/AADC−, TH−/AADC+, and TH+/AADC+—were found at all levels of the PeVN in the rostro-caudal axis, but the number of neurons in these populations at different levels of the PeVN differed significantly (Figure 7). Thus, throughout the PeVN in the rostro-caudal direction (240–960 μm), except for the most caudal level (1200 μm), TH−/AADC+ neurons quantitatively dominated. At three rostral levels of the PeVN (240–720 μm), the number of TH+/AADC− and TH+/AADC+ neurons was approximately the same, but was many times lower than the number of TH−/AADC+ neurons. At caudal levels three to six (720–1200 μm), the number of TH+/AADC− and TH+/AADC+ neurons increased, while the number of TH−/AADC+ neurons decreased. At the most caudal level of the PeVN, TH+/AADC− neurons predominated, while the numbers of TH−/AADC+ and TH+/AADC+ neurons became equal (Figure 7).

## 3. Discussion

**Figure 8:**
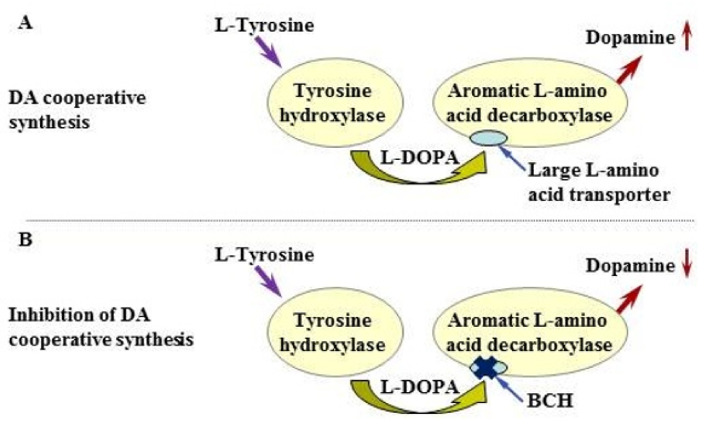
Schematic representation: (**A**) cooperative synthesis of dopamine (DA) by monoenzymatic neurons containing one of the enzymes of dopamine synthesis—tyrosine hydroxylase or aromatic L-amino acid decarboxylase; (**B**) inhibition of cooperative DA synthesis by 2-amino-2-norbornanecarboxylic acid (BCH), an inhibitor of the large neutral amino acid membrane transporter.

From the late 1980s, after the development of TH mono-immunolabeling, until the early 2000s, TH-immunopositive neurons found in dopamine-rich areas of the brain were considered to be dopaminergic neurons. However, it was later shown that (i) in addition to dopaminergic neurons, most of these brain regions contain numerous neurons expressing single enzymes of dopamine synthesis, TH or AADC [6,7,8,9,23,28]; (ii) monoenzymatic TH- and AADC-expressing neurons synthesize dopamine in cooperation [16,17] (Figure 8A); (iii) L-DOPA, the final synthetic product in monoenzymatic TH neurons [5,10,11], in addition to participating in the cooperative synthesis of dopamine, plays the role of a neurotransmitter in adulthood and, apparently, a morphogenetic (transcriptional) factor in ontogenesis [9,18,19,34]; (iv) the cooperative synthesis of dopamine by monoenzymatic neurons is a compensatory mechanism during the degeneration of dopaminergic neurons in the brain and following spinal cord injury [9,15,28].

In the last 20 years, it was shown using double immunolabeling for TH and AADC that monoenzymatic neurons are widely distributed in the brain, in the spinal cord, and in the peripheral nervous system [7,9,28]. This study aimed to prove that PeVN is also a prominent dopaminergic center containing not only bienzymatic (dopaminergic) neurons, but also monoenzymatic TH and AADC neurons. This nucleus of the hypothalamus has so far been well studied from the point of view of its participation in the regulation of reproduction [12,35,36,37], but it has never been considered as a dopaminergic center, containing numerous dopaminergic (bienzymatic) and monoenzymatic neurons. Nevertheless, in rare studies, monoenzymatic TH neurons located in the PeVN have been described. However, the functional role of TH in these neurons has not been considered [12,38]. Surprisingly, in a number of papers focusing on the PeVN and some other brain areas, the detection of only TH in neurons is still considered sufficient to identify them as dopaminergic neurons [30,35].

When starting this study, we had to define the boundaries of the PeVN, since they vary somewhat in different papers [39]. We consider the PeVN to be a periventricular zone on both sides of the third cerebral ventricle up to 300 μM wide, which extends rostro-caudally from the optic chiasm to the rostral AN, and is located ventrodorsally at the level of the middle third of the third ventricle. It should be noted that the PeVN area does not include the dorsally located paraventricular nucleus, the ventrally located suprachiasmatic nucleus, or the medial preoptic area.

We studied the PeVN in rats at the age of 30 days, i.e., at a time when, according to the existing research literature, the main processes of “maturation” of the brain, including the formation of neuronal networks and synaptogenesis, as well as the closure of the blood–brain barrier, have already been completed [40,41,42]. In a number of experiments, for comparative analysis and/or as control, we used the AN as a well-studied dopaminergic center containing dopaminergic neurons and monoenzymatic neurons, as well as the substantia nigra (compact part) as a dopaminergic center containing predominantly dopaminergic neurons [24].

**Figure 9:**
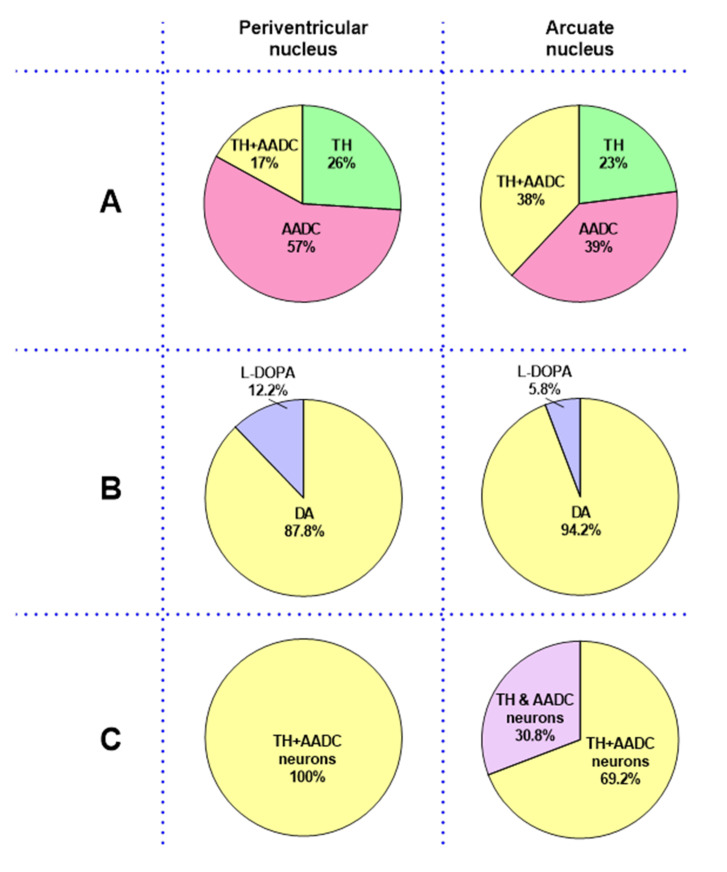
Comparative analysis of the periventricular nucleus (PeVN) and the arcuate nucleus (AN) as brain centers containing neurons expressing one or both dopamine (DA)-synthesizing enzymes in rats aged 30 days: (**A**) proportions of monoenzymatic (TH or AADC) and bienzymatic (TH + AADC) neurons in the PeVN and AN (Ershov et al., 2002) in relation to all neurons expressing enzymes of DA synthesis, taken as 100%; (**B**) proportions of DA and L-DOPA in each nucleus in relation to the total content of each substance, taken as 100%; (**C**) proportions of DA synthesis by bienzymatic neurons (TH + AADC) and monoenzymatic neurons (TH & AADC) in cooperation in the PeVN and AN in relation to the total synthesis of DA, taken as 100%. AADC, aromatic L-amino acid decarboxylase; TH, tyrosine hydroxylase.

The first and most important evidence for our hypothesis about the PeVN as a dopaminergic center containing dopaminergic and non-dopaminergic monoenzymatic neurons is the discovery that this nucleus in rats contains numerous neurons which are immunopositive only for TH, only for AADC, or for both enzymes of dopamine synthesis, comprising about three thousand neurons in total. According to these data, the PeVN differs little from the AN, having about three and a half thousand such neurons [9]. More precisely, the number of neurons expressing the enzymes of dopamine synthesis in the PeVN and AN differs by less than 16%. A more detailed comparative quantitative analysis of each of the three populations of neurons in the PeVN (see Results) and in the AN [15] showed that monoenzymatic AADC neurons predominate in both nuclei, and their numbers and proportions are greater in the PeVN than in the AN. As for the proportions of monoenzymatic TH neurons and bienzymatic neurons in the PeVN and in the AN, they differ significantly. In fact, the proportion of monoenzymatic TH neurons in the PeVN is much larger, whereas the proportion of bienzymatic neurons is significantly lower than in the AN (Figure 9, upper layer).

Given the significant rostro-caudal extension of the PeVN, which was approximately 1.2 mm in rats aged 30 days, data on the content of bienzymatic and monoenzymatic neurons at various frontal levels of this nucleus are of particular interest. The quantification of bienzymatic and monoenzymatic neurons on serial frontal sections of the PeVN showed that monoenzymatic AADC neurons strongly predominate in the nucleus over 960 μm in the rostro-caudal extension. Only in the most caudal part of the PeVN, 240 µm long, did the number of AADC neurons decrease by 1.5 times. The distribution of bienzymatic and monoenzymatic TH neurons along the rostro-caudal axis of the PeVN differs significantly from that of monoenzymatic AADC neurons. Indeed, in the rostral part of the nucleus, at a distance of 720 μm in the caudal direction, the number of bienzymatic and monoenzymatic TH neurons is approximately the same and 4.5 times lower than the number of monoenzymatic AADC neurons in the same area. Over the next 480 µm, up to the caudal end of the PeVN, the number of bienzymatic and monoenzymatic TH neurons progressively increases. In this area of the PeVN, monoenzymatic TH neurons predominate among all three studied populations. The data obtained suggest that if L-DOPA is synthesized in the monoenzymatic TH neurons as the final synthetic product, and dopamine is synthesized in monoenzymatic AADC neurons and bienzymatic neurons, then the efficiency of these syntheses should be the highest in the caudal part of the PeVN.

In order to answer the question of whether L-DOPA and dopamine are synthesized in the neurons of the PeVN, these substances were measured using high performance liquid chromatography (HPLC). The PeVN was shown to contain L-DOPA and much higher levels of dopamine. We found the same content of L-DOPA in the PeVN as in the AN, whereas the content of dopamine in the PeVN was slightly lower than in the AN (Figure 9, middle layer). Given the fact that AADC activity in dopaminergic neurons usually greatly exceeds TH activity, and therefore L-DOPA is not detected in these neurons or is detected in trace amounts, it is assumed that L-DOPA, detected by HPLC in the PeVN and AN, is the final synthetic product in TH monoenzymatic neurons. Indeed, it was previously shown using immunocytochemistry that L-DOPA in the AN is contained in monoenzymatic TH neurons, whereas dopamine is an attribute of monoenzymatic AADC neurons and bienzymatic neurons [5,8,10,11].

When evaluating the metabolic activity of monoenzymatic and bienzymatic neurons in the PeVN, it was necessary to answer the question as to whether dopamine, which was found in this nucleus, is synthesized only in the dopaminergic neurons or, as in the AN, whether it is also synthesized by monoenzymatic neurons containing TH or AADC, in cooperation (Figure 8A). In order to answer this question, we used the same experimental design which allowed us in the past to obtain evidence of cooperative dopamine synthesis by monoenzymatic neurons in the AN [16,17]. In this experiment, a cell suspension of the AN of fetal rats [16] or vibratome sections of the AN of adult rats [17] were incubated in the presence of large neutral L-amino acids, which competitively inhibit L-type amino acid transporter 1 (LAT1) of these amino acids and L-DOPA from the intercellular environment into cells, including monoenzymatic AADC neurons [16,17]. Evidence of the cooperative synthesis of dopamine included a decrease in the total dopamine content in the incubation medium and in the AN cell suspension [16] or in the incubation medium and in the vibratome sections of AN [17] after incubating cells or vibratome sections with large neutral L-amino acids in a high concentration, compared to the control (incubation of cells or sections in an incubation medium without large neutral L-amino acids).

To test the idea of cooperative dopamine synthesis by monoenzymatic neurons in the PeVN, vibratome sections of this nucleus were incubated in the same way as the AN vibratome sections in our previous study [17]. However, to inhibit L-DOPA uptake by AADC monoenzymatic neurons, instead of large neutral L-amino acids, we used BCH with a much higher affinity for LAT1 (Figure 8B) [43,44]. In the routine control, PeVN vibratome sections were incubated without BCH. As an additional physiological control, AN vibratome sections obtained from rats aged 30 days were incubated under the same conditions as PeVN vibratome sections. As expected, the incubation of AN vibratome sections with BCH led to a decrease in the total dopamine content in the incubation medium and in the sections when compared with the control (incubation without BCH). However, the incubation of PeVN vibratome sections in the presence of BCH did not lead to a change in the total dopamine content in the incubation medium and in the sections when compared with the control (incubation without BCH). These data show that there is no cooperative synthesis of dopamine by monoenzymatic neurons in the PeVN, in contrast to the AN (Figure 9, bottom layer). One of the reasons for the lack of cooperative synthesis of dopamine in the PeVN may be the absence in this nucleus of the same close topographic relationships between monoenzymatic TH neurons and AADC neurons, as in the AN, which is crucial for the effective transfer of L-DOPA from one neuron to another. Indeed, using confocal and electron microscopy, we previously demonstrated that in the AN there were direct contacts between monoenzymatic TH neurons and monoenzymatic AADC neurons, the most numerous being in the median eminence, the main site for axonal projections of monoenzymatic neurons of this nucleus [17,22].

Despite the absence of cooperative synthesis in the PeVN, we have shown, using confocal microscopy, that some monoenzymatic neurons are in topographic proximity to each other. Of particular interest are our observations of direct contacts between monoenzymatic neurons at the level of cell bodies and their proximal processes, on the one hand, and monoenzymatic nerve fibers containing TH or AADC, on the other hand. These data suggest that the monoenzymatic neurons of the PeVN, as those of the AN, synthesize dopamine in cooperation, but at a much lower level that is undetectable by HPLC. In addition to the close topographic relationships between monoenzymatic neurons, we found the same close contacts of monoenzymatic neurons with bienzymatic neurons and with nerve fibers detected by a mixture of antibodies to tryptophan hydroxylase and dopamine-beta-hydroxylase. The latter can only belong to brainstem serotonergic neurons and/or noradrenergic neurons. Taken together, these data suggest that the monoenzymatic and bienzymatic neurons of the PeVN are under complex control both from the neurons of the PeVN itself and from other hypothalamic and extrahypothalamic monoaminergic centers of the brain.

Our confocal microscopic data on the close topographic relationships of bienzymatic and monoenzymatic neurons (cell bodies) and nerve fibers with the third ventricle are also of great interest. Indeed, monoenzymatic and bienzymatic fibers were found in the lumen of the ventricle, between the ependymal cells and in the subependymal region, while neurons (cell bodies) are located in the subependymal region only. We believe that dopamine and L-DOPA, released from bienzymatic and monoenzymatic neurons and fibers, are delivered to the third ventricle either directly from supra-ependymal fibers or diffusely from the sub-ependymal region through the ependymal lining. This idea was indirectly supported by the fact that we detected dopamine and L-DOPA in the CSF using HPLC in rats of the same age. No less interesting is the fact that the concentration of L-DOPA in the CSF significantly exceeded the concentration of dopamine. Considering that the concentration of both substances in the CSF is high enough to affect target cells, it is quite possible that they play the role of neurohormones involved in the neuroendocrine regulation of brain target neurons. The above data obtained in our study of the PeVN are consistent with previous studies, which also showed the ingrowth of axons of monoaminergic and monoenzymatic neurons into the cerebral ventricles. These studies also suggest that physiologically active substances delivered to the CSF thanks to axo-ventricular contacts play the role of neurohormones that provide volume neurotransmission [3,27,45,46].

Considering the functional significance of monoenzymatic neurons, one should take into account the fact that many of them are peptidergic. The enzymes of dopamine synthesis in these neurons are permanently or transiently co-expressed with peptides. For example, AADC is permanently co-expressed with vasopressin in neurons of the suprachiasmatic nucleus [47], whereas TH is transiently co-expressed with the same peptide in the supraoptic and paraventricular nuclei upon chronic osmotic stimulation [48]. Moreover, it has been shown that TH is co-expressed with kisspeptin in the PeVN and AN neurons [12,30,38]. Surprisingly, despite the fact that there are many examples of neurons co-expressing neuropeptides and dopamine-synthesizing enzymes, only a few studies considered the possibility of their co-participation in neurotransmission [38]. Stephens et al. (2017) [13] attempted to elucidate the functional role of TH in kisspeptin-producing neurons of the AN and PeVN, which play a key role in the regulation of reproduction. However, the switching off of this enzyme in kisspeptin neurons did not cause pronounced changes in the reproductive function.

Earlier studies of PeVN did not focus on neurons expressing dopamine-synthesizing enzymes. On the contrary, this study opens up broad prospects for evaluating the functioning, functional significance, and regulation of these neurons. By analogy with other brain centers containing bienzymatic and monoenzymatic neurons, in the future it is important to assess: (i) what is the regulatory role of the end products of synthesis—L-DOPA and dopamine, within and outside the PeVN; (ii) whether the cooperative synthesis of dopamine by monoenzymatic TH and AADC neurons is activated under systemic failure of the dopaminergic systems of the brain, primarily in Parkinson’s disease; (iii) whether the expression of TH and AADC is associated with the kisspeptin regulation of reproduction.

## 4. Materials and Methods

### 4.1. Animals

**Figure 10:**
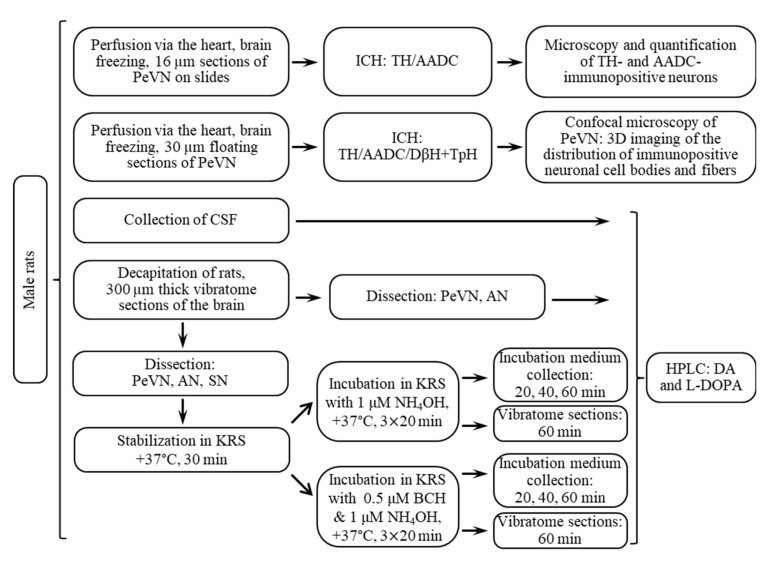
Scheme of experiments carried out on male Wistar rats at the age of 30 days. AADC, aromatic L-amino acid decarboxylase; AN, arcuate nucleus; BCH, 2-amino-2-norbornanecarboxylic acid; DA, dopamine; DβH, dopamine β hydroxylase; L-DOPA, L-3,4-dihydroxyphenylalanine; HPLC, high-performance liquid chromatography; ICH, immunocytochemistry; KRS, Krebs-Ringer solution; NH_4_OH, ammonium hydroxide; PeVN, periventricular nucleus; TpH, tryptophan hydroxylase; TH, tyrosine hydroxylase; SN, substantia nigra.

The research was carried out on male Wistar rats on the 30th postnatal day weighing 85–110 g (*n* = 136) (Figure 10). The animals were kept under standard vivarium laboratory conditions: at a temperature of 21–23 °C, with a 12-h day-night routine, with free access to food and water.

All manipulations with the animals were performed in accordance with the requirements of the National Institutes of Health (NIH Guide for the Care and Use of Laboratory Animals) and the Bioethics Committee of the Koltzov Institute of Developmental Biology named after N.K. Koltsov (Protocol No. 44 dated 24 December 2020 and Protocol No. 55 dated 9 December 2021).

### 4.2. Experiments and Obtaining Samples for Analysis

#### 4.2.1. Cerebrospinal Fluid Collection

Rats (*n* = 50) were given isoflurane anesthesia (Laboratorios Karizoo, Barcelona, Spain) using an anesthesia device, SomnoSuite (Kent Scientific, Torrington, CT, USA). After that, cerebrospinal fluid (CSF) from the cisterna magna was obtained from rats according to a previously described method [49]. For this, the head of the animal was fixed in stereotaxis (Narishige Lab, Tokyo, Japan) and access to the cisterna magna was provided. A glass microcannula connected with a Teflon tube to a Hamilton syringe was stereotactically inserted into the cisterna magna. From each rat, we received 50 ± 20 μL CSF. The collected CSF was immediately supplemented with HClO_4_, at a final concentration of 0.1 N, and using a 5 pmol internal standard 3,4-dihydroxybenzylamine hydrobromide (DHBA) (Sigma, Saint Louis, MO, USA). Thereafter, CSF samples were frozen in liquid nitrogen and stored at −70 °C until dopamine and L-DOPA were determined by HPLC (Figure 10).

#### 4.2.2. Preparation of Vibratome Brain Sections and Their Incubation

**Figure 11:**
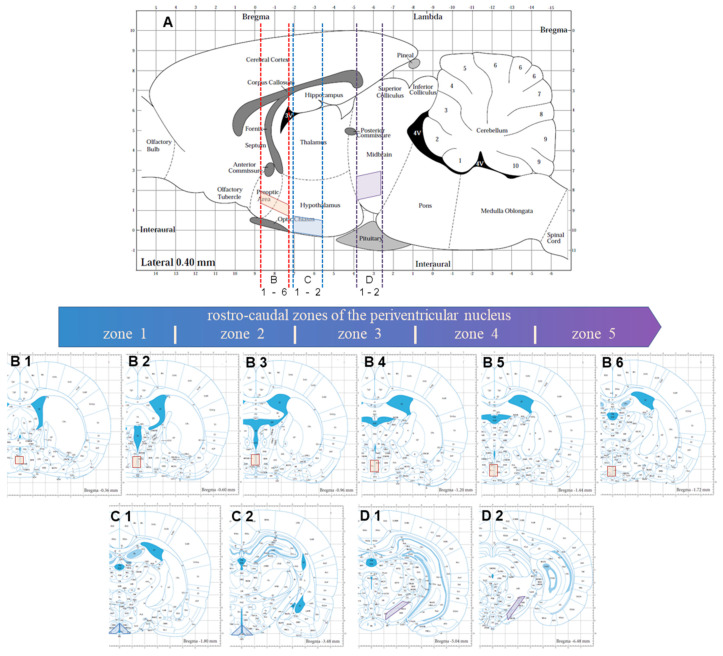
Scheme of obtaining frontal vibratome sections, 300 μm thick, of the brain in the rostro-caudal direction at a frontal level corresponding to the periventricular nucleus (PeVN) with conditionally allocated zones (**B**) 1–6, as well as the arcuate nucleus (AN) with zones (**C**) 1,2, and the substantia nigra (ES) with zones D1,2, according to the rat brain steriotaxic atlas [50]. (**A**) the sagittal plane of the brain; (**B**–**D**) the frontal planes of the brain; red frames, borders of the selected brain regions: PeVN, AN, and SN.

Rats (*n* = 60) under isoflurane anesthesia were decapitated and their brains were removed. Then, serial 300 µm thick frontal sections were prepared on a vibratome (Leica VT 1200S, Leica, Wetzlar, Germany) in Krebs–Ringer solution (KRS) (mM: NaCl 120, KCl 4.8, CaCl_2_ 2, MgSO_4_ 1.3, NaHCO_3_ 25, d-glucose 10, HEPES 20, ascorbic acid 0.1; pH 7.4, 4 °C). For subsequent incubations, 4 sections were selected at the level of the PeVN, AN, and the substantia nigra (SN), according to the rat brain atlas [50], adjusted for age. The PeVN corresponded to the frontal sections obtained along the coordinates from −0.36 mm to −1.72 mm from the bregma in the rostro-caudal direction, and in the dorso-ventral direction at the level of the middle third of the third ventricle (Figure 11). Frontal AN sections were obtained in the rostro-caudal direction at the coordinates from −1.8 mm to −3.48 mm from the bregma [50], and in the dorso-ventral direction at the level of the lower third of the third ventricle (Figure 11). Frontal SN sections were obtained in the rostro-caudal direction at the coordinates from −5.04 mm to −6.48 mm from the bregma [50] (Figure 11). After that, with each frontal section of the brain under the control of a binocular microscope (Leica M60, Germany), the corresponding area of the brain (PeVN, AN, SN) was cut out with a blade. The resulting sections were divided into two halves, which were symmetrical with respect to the midsagittal plane.

For static incubation, each 1 mL thermostatic chamber containing 500 μL KRS (pH 7.4) was loaded with PeVN, AN, or SN sections obtained from 2 rats from one half of the brain. Sections of each of the brain regions studied (PeVN, AN, SN) were incubated in 20 chambers at 37 °C. In the first 30 min there was a stabilization of sections. Then, in half of the chambers (10 chambers per brain region), KRS was replaced with KRS containing 0.5 mM BCH, an inhibitor of LAT1 [51]. As recommended by the manufacturer (Catalog A7902, Sigma, Saint Louis, MO, USA), BCH was dissolved in 10% aqueous NH_4_OH and then diluted 250-fold in KRS. The sections were incubated for 1 h at 37 °C, collecting successively three 20-min fractions of the incubation medium and replacing the incubation medium with a fresh one. Each experiment was carried out 3–4 times. For the control (10 chambers per brain region), PeVN, AN, and SN vibratome sections obtained from the same rats from the other half of the brain were incubated. In this case, the sections were incubated in KRS without BCH, supplemented with 0.04% NH4OH (Figure 10). In each experiment, three 20-min incubation medium fractions from the incubation of PeVN, AN, or SN sections were pooled and 100 μL 1 N HClO_4_ and 2 pmol DHBA were added per 900 μL of incubation medium. All the obtained medium samples were frozen in liquid nitrogen. After the end of the incubation, the sections were weighed and also frozen in liquid nitrogen. The frozen samples were stored at −70 °C until the HPLC determination of dopamine content.

#### 4.2.3. Obtaining Samples of Periventricular and Arcuate Nucleuses for Biochemical Analysis

Rats (*n* = 20) under isoflurane anesthesia were decapitated and the brain was isolated. PeVN and AN samples were then obtained from the brain according to the method described in Section 4.2.2. PeVN or AN vibratome sections obtained from both halves of the brain were used as one sample. The sections were frozen in liquid nitrogen and stored at −70 °C until dopamine and L-DOPA content was determined by HPLC.

#### 4.2.4. Preparing the Brain for Immunohistochemistry

For 15 min, rats (*n* = 6) under chloral hydrate anesthesia (ip, 400 mg/kg) were perfused through the heart with 0.9% NaCl in 0.02 M phosphate buffer (phosphate-buffered saline, PBS) (pH = 7.2–7.4) at +37 °C, followed by 15 min 4% paraformaldehyde in 0.1 M phosphate buffer (pH 7.2–7.4) at +4 °C. After that, the animals were decapitated, and their brains were isolated. The brains were additionally fixed by immersion in 4% paraformaldehyde for 12 h at +4 °C, and then washed in PBS and incubated in 20% sucrose at 0.02 M PBS at +4 °C for 24 h. The brains were then frozen at –40 °C and stored at −70 °C until immunocytochemistry was performed.

### 4.3. Methods

#### 4.3.1. High-Performance Liquid Chromatography

Frozen CSF samples from one animal were thawed. After that, the CSF from 9–11 rats was combined into one sample. The received CSF samples with a volume of approximately 500 μL were processed by solid-phase extraction, adding alumina (15 mg per sample) (Sigma, Saint Louis, MO, USA) and 1.5 M Tris buffer (pH 8.6) at 4 °C and shaking for 15 min in the darkness. The final solution was centrifuged at 1700× *g* for 3 min and 4 °C, and the supernatant was removed. The precipitate was washed twice with bi-distilled water for 3 min, followed by centrifugation at 1000× *g* and 4 °C for 3 min and the removal of the supernatant. The elution of catecholamines was performed by adding 120 μL of 0.2 N HClO_4_ and the suspension was centrifuged for 15 min at 1000× *g* and 4 °C. Then, the supernatant was collected, for which the content of dopamine and L-DOPA was determined.

Frozen nervous tissue samples were thawed and homogenized in 120 μL 0.1 N HClO_4_ with 10 pmol/mL DHBA using an ultrasonic homogenizer (UP100H, Hielscher, Teltow, Germany). In the obtained samples, the protein content was determined by the previously described BCA Protein Assay method [52]. The sample homogenates were then centrifuged for 15 min at 20,000 g and 4 °C and the supernatant was collected, in which the dopamine and L-DOPA content was determined.

Dopamine and L-DOPA were determined on a reversed-phase column ReproSil-Pur, ODS-3, 4 × 100 mm with a pore diameter of 3 µm (Dr. Majsch, Entringen, Germany), at a temperature of +28 °C and a mobile phase speed of 1 mL/min, supported by a liquid chromatograph LC-20ADsp (Shimadzu, Kyoto, Japan) at 850 mV. The mobile phase consisted of 0.1 M citrate–phosphate buffer, 0.3 mM sodium octanesulfonate, 0.1 mM EDTA, and 8% acetonitrile (all reagents from Sigma, Saint Louis, MO, USA) (pH 2.58). Dopamine and L-DOPA were determined using a fluorescent detector, RF-20A (Shimadzu, Kyoto, Japan), at a wavelength of 285/316 nm. The peaks of the substances were identified by the time of their release into the standard solution.

#### 4.3.2. Immunohistochemistry of Sections on Slides

For an immunocytochemical study of PeVN neurons expressing dopamine synthesis enzymes, serial frontal sections were prepared in the rostro-caudal direction starting from −0.36 mm to −1.72 mm from the bregma [50], with a 16 μm thickness using a cryostat (Leica CM1950, Leica, Wetzlar, Germany). The PeVN sections were mounted on slides and successively incubated in PBS containing: (a) 1% sodium lauryl sulfate (Sigma, Saint Louis, MO, USA) for 5 min at +20 °C; (b) 5% bovine serum albumin (Sigma, Saint Louis, MO, USA) and 0.3% Triton X-100 (Sigma, Saint Louis, MO, USA) for 1 h at +20 °C; (c) mouse monoclonal antibodies to TH (1:900) (Sigma, Saint Louis, MO, USA), rabbit polyclonal antibodies to AADC (1:300) (Abcam, Cambridge, UK), 1% bovine serum albumin, and 0.1% Triton X-100 for 20 h at +20 °C; (d) goat Alexa-Fluor-546 antibodies against rabbit gamma globulins (1:1000) (Invitrogen, Waltham, MA, USA) for 2 h at +20 °C; (e) donkey Alexa-Fluor-488 antibody against mouse gamma globulins (1:1000) (Invitrogen, Waltham, MA, USA) for 2 h at +20 °C. After each incubation, except for the last one, the sections were washed in PBS 3 times for a total of 45 min. After the last incubation, the sections were washed in PBS for an hour and embedded in a medium containing DAPI (Abcam, Cambridge, UK), a dye for cell nuclei.

#### 4.3.3. Immunohistochemistry of Floating Sections

For an immunocytochemical study of the interneuronal and axo-ventricular contacts of PeVN neurons expressing dopamine synthesis enzymes, PeVN frontal serial sections 30 µm thick were prepared using a cryostat (Leica CM1950, Leica, Wetzlar, Germany). Every 8th section was taken in the rostro-caudal direction starting from −0.36 mm to −1.72 mm from the bregma [50] and was placed in PBS (“floating sections”).

The floating sections were successively incubated with: (a) 1% sodium lauryl sulfate (Sigma, Saint Louis, MO, USA) for 5 min at +20 °C; (b) 3% bovine serum albumin (Sigma, Saint Louis, MO, USA) and 0.3% Triton X-100 (Sigma, Saint Louis, MO, USA) for 1 h at +20 °C; (c) rabbit polyclonal antibodies to AADC (1:300) (Abcam, Cambridge, UK), sheep polyclonal antibodies to TH (1:700) (Sigma, Saint Louis, MO, USA), 1% bovine serum albumin, and 0.1% Triton X-100 for 20 h at +20 °C; (d) monoclonal mouse antibodies to dopamine β hydroxylase (DβH) (1:300) (Sigma, Saint Louis, MO, USA), mouse antibodies to tryptophan hydroxylase (TpH) (1:1000) (Sigma, Saint Louis, MO, USA), 1% bovine serum albumin, and 0.1% Triton X-100 for 20 h at +20 °C; (e) biotinylated goat antibodies against rabbit gamma globulins (1:200) (Vector Laboratories, Newark, CA, USA) for 2 h at +20 °C; (f) streptavidin–Cy3 (1:100) (Sigma, Saint Louis, MO, USA) for 1 h at +20 °C; (g) donkey Alexa-Fluor 633 antibodies against sheep gamma globulins (1:1000) (Invitrogen, Waltham, MA, USA) and Alexa-Fluor 488 donkey antibodies against mouse gamma globulins (1:1000) (Invitrogen, Waltham, MA, USA) for 2 h at +20 °C;. All the solutions were prepared on the basis of PBS, which was also used for washing the sections between the incubations for 30 min, as well as for 1 h after the last incubation. The sections were then mounted on slides and embedded in a medium containing DAPI (Abcam, Cambridge, UK).

#### 4.3.4. Microscopy of Periventricular Nucleus Thin Sections on Slides and Quantification of Neurons Expressing Dopamine Synthesis Enzymes

Thin brain sections, including PeVN, after double immunostaining for TH and AADC, were examined using a fluorescence microscope, Zeiss Observer Z1 (Zeiss, Oberkochen, Germany), with a 20× objective magnification. To create a panoramic image, the “mosaic” function (AxioVision 4.8 software, Zeiss, Oberkochen, Germany) was used, so that the entire 3rd ventricle and 300 μm of nervous tissue on each side of the ventricle were visible on the final image. Next, the number of neurons that were immunopositive for one or two enzymes (TH and/or AADC), as well as the total number of these neurons located in the PeVN—in the periventricular zone along the 3rd ventricle up to 300 μm wide—were counted (Figure 11). Next, in the selected area, the number of neurons with a visible cell nucleus was counted. The Abercrombie test [53] was used to exclude the double counting of neurons located on neighboring sections:

$$N=\frac{n*\Delta }{\Delta +d}$$

N—the total number of cells in a section

n—the number of counted cells in a section,

Δ—section thickness (16 μm)

d—average cell nucleus diameter

In addition to the number of neurons which are TH-immunopositive only, AADC-immunopositive only, or both TH-immunopositive and AADC-immunopositive, we determined the proportion of each of these populations as a percentage of the total number of all neurons, taken as 100%, using the formula:

$$P_{n}=\frac{N_{n}}{N_{\mathrm{TH}}+N_{\mathrm{AADC}}+N_{\mathrm{TH}/\mathrm{AADC}}}*100\%$$

P_n_—percentage of neurons which are TH-immunopositive only, AADC-immunopositive only, or both TH-immunopositive and AADC-immunopositive

N_n_—number of neurons which are TH-immunopositive only, AADC-immunopositive only, or both TH-immunopositive and AADC-immunopositive

N_TH_—number of TH-immunopositive neurons

N_AADC_—number of AADC-immunopositive neurons

N_TH/AADC—_number of neurons which are both TH-immunopositive and AADC-immunopositive

To assess the distribution of neurons, the PeVN area was divided into 5 zones in the rostro=caudal direction: each zone was 240 μm long (15 sections 16 μm thick) (Figure 11). In each PeVN zone (on 15 consecutive serial frontal sections), the number of neurons with a visible cell nucleus was counted for each type of neuron (mono- and bienzymatic) in the same way as described for the entire PeVN.

#### 4.3.5. Confocal Microscopy of the Periventricular Nucleus, Thick Floating Sections and 3D Analysis of the Neuron Topography: Cell Bodies and Processes Containing Dopamine Synthesis Enzymes

Brain sections containing PeVN, after triple immunolabeling for TH, AADC, DβH, and TpH were examined with a confocal microscope, Zeiss LSM880 (Zeiss, Oberkochen, Germany), using the objectives Plan-apochromat 20×/0.8 M2 and Plan-Apochromat 63×/1.40 Oil DIC M27. Photographs were taken in two channels; in the first channel, the signals from AlexaFluor 633 and AlexaFluor 488 were simultaneously detected, in the second channel, those from AlexaFluor 546 and DAPI were detected. A panoramic shot was taken using the “mosaic” function 1 × 4 with z-stack (optical section 2.5 μm thick) at a 20× magnification. To assess the topographic relationships between the bodies of neurons and nerve fibers, a z-stack (optical section thickness 0.6 μm) was made at a magnification of 63× with an additional 2 x zoom. Image processing was done in ZenBlue (Zeiss, Oberkochen, Germany).

### 4.4. Statistical Analysis

Data are presented as mean ± standard error of mean (mean ± SEM). The correspondence of the data to the normal distribution was checked using the Shapiro–Wilk test. The results were statistically processed with the GraphPad Prism 6.0 software package (GraphPad Software, San Diego, CA, USA), using one-way ANOVA, the parametric Student’s *t*-test, or the non-parametric Mann–Whitney U test. *p* ≤ 0.05 was used everywhere as a significance criterion.

## 5. Conclusions

It was shown that:

(1) The PeVN of the hypothalamus is one of the powerful dopaminergic centers in the brain, which contains numerous neurons expressing both or individual complementary enzymes of dopamine synthesis;
(2) L-DOPA and dopamine are produced in the PeVN as the final synthetic products, most probably in monoenzymatic TH neurons and monoenzymatic AADC neurons and/or bienzymatic neurons;
(3) Bienzymatic and monoenzymatic neurons (cell bodies and fibers) are topographically closely related to each other, as well as to the third ventricle, providing L-DOPA and dopamine delivery pathways to the CSF.

## Data Availability

The data presented in this study are available on request from the corresponding author. The data are not publicly available due to legal issues.

## Abbreviations

AADC	aromatic L-amino acid decarboxylase
AN	arcuate nucleus
BCH	2-amino-2-norbornanecarboxylic acid
CSF	cerebrospinal fluid
HPLC	high performance liquid chromatography
KRS	Krebs-Ringer solution
LAT1	L-type amino acid transporter 1
PD	Parkinson’s disease
PeVN	periventricular nucleus
TH	tyrosine hydroxylase
TPH	tryptophan hydroxylase
